# Is delaying pharmacological thromboprophylaxis associated with thromboembolic complications?

**DOI:** 10.1186/cc14403

**Published:** 2015-03-16

**Authors:** P Padim, A Alshafai, S Canestrini, S Rizoli, J De Rezende Neto, A McFarlan

**Affiliations:** 1 Universidade de São Paulo, Ribeirao Preto, Brazil; 2 St Michael's Hospital, Toronto, ON, Canada; 3 St George's Hospital, London, UK

## Introduction

Thromboembolic complications (TEC) are very common and lethal in patients suffering from traumatic injury [1]. The trauma clinical guidelines recommend the administration of pharmacological thromboprophylaxis (PTP) to reduce the risk of developing TEC [2]. However, it is unknown whether delayed PTP initiation increases risk of TEC. We hypothesize that delayed PTP initiation is associated with increased TEC rates.

## Methods

A retrospective chart review (2010 to 2013) was conducted on adult trauma patients that were admitted into a level 1 Trauma Centre in Toronto. Demographics, date of PTP initiation, date of TEC diagnosis (CT-PE/US Doppler), injury type and severity were collected. A comparison between early and late PTP initiation has been made with regards to TEC development. Student's *t *test, univariate and multivariate logistic regression analyses were performed.

## Results

A total of 1,312 patients received PTP, 821 (62.5%) initiated early PTP (within 48 hours) while 491 (37.5%) initiated after 48 hours. The group that initiated early prophylaxis was younger (mean: 46 vs. 55, *P *< 0.0005), had lower ISS (mean: 17 vs. 24, *P *< 0.0005), shorter length of stay (LOS) (mean: 11 vs. 23, *P *< 0.0005), more pelvic fractures (19% vs. 13%, *P *= 0.0058), more head injury (AIS Head ≥3, *P *< 0.0005), less blunt trauma (85% vs. 95%, *P *< 0.0005), lower incidence of TEC (5.3% (44) vs. 8.5% (42), *P *= 0.023), and lower mortality rate (1.5% vs. 7.5%). Univariate analysis showed LOS (*P *< 0.0005), ISS (*P *< 0.0005), time to PTP initiation (*P *= 0.0018) and blunt MOI (*P *= 0.0099) significantly associated with TEC events. Multivariate analysis, however, showed TEC events correlated only to LOS (*P *= 0.0001). Stepwise multiple logistic regression confirmed LOS as independently associated with TEC events (95% CI = 0.003, 0.006, *P *< 0.0005).

## Conclusion

Mortality rates in patients with delayed PTP are higher. Our study shows LOS as the only independent predictor for TEC. However, this might not necessarily reflect causation. Delayed PTP appears not to be an independent predictor to TEC events in trauma patients, which favours current clinical trends when it comes to contraindicating early PTP initiation.
